# Correlation analysis between expression of histone deacetylase 6 and clinical parameters in IgA nephropathy patients

**DOI:** 10.1080/0886022X.2021.1914657

**Published:** 2021-04-26

**Authors:** Yan Hu, Minghua Shang, Yingfeng Shi, Min Tao, Weijie Yuan, Lunxian Tang, Xiaoyan Ma, Binbin Cui, Hui Chen, Xun Zhou, Shougang Zhuang, Na Liu

**Affiliations:** a Department of Nephrology, Shanghai East Hospital, Tongji University School of Medicine, Shanghai, China; b Department of Nephrology, Shanghai General Hospital, Shanghai Jiao Tong University School of Medicine, Shanghai, China; c Emergency Department of Critical Care Medicine, Shanghai East Hospital, Tongji University School of Medicine, Shanghai, China; d Department of Medicine, Rhode Island Hospital and Alpert Medical School, Brown University, Providence, RI, USA

**Keywords:** IgA nephropathy, histone deacetylase 6, acetyl histone H3, renal dysfunction, Oxford Classification

## Abstract

**Background:**

It has been demonstrated that histone deacetylase 6 (HDAC6) is involved in various kidney diseases in experimental study. However, correlation between HDAC6 and clinical parameters in IgA nephropathy (IgAN) patients is still unknown.

**Methods:**

A total of 46 human kidney biopsy specimens with IgAN were selected as observation group, specimens of normal renal cortex tissue that was not affected by the tumor from patients with renal carcinoma (*n* = 7) served as control. We investigated the relationship between HDAC6 and clinical parameters in IgAN.

**Results:**

HDAC6 was highly expressed in human kidney biopsy specimens with IgAN compared with control group, while the number of acetyl histone H3 positive cells were significantly decreased. There was a statistical difference in the indexes of albumin, estimated glomerular filtration rate (eGFR), serum urea, serum creatinine, serum uric acid, β2-microglobulin, cystatin C, cholesterol, high-density lipoprotein, low-density lipoprotein, and HDAC6 positive area among the different Oxford Classification (*p* < 0.05). The expression of HDAC6 was different in various eGFR levels, the expression of HDAC6 increased with the decreasing of eGFR level, the expression of acetyl histone H3 decreased with the decreasing of eGFR level. In addition, the expression of HDAC6 positively correlated with Masson trichrome positive area, serum urea, serum creatinine, β2 macroglobulin, and cystatin C, while negatively correlated with eGFR and acetyl histone H3. Multivariate linear regression analysis demonstrated that eGFR and cystatin C were independently associated with HDAC6, respectively (*p* < 0.05).

**Conclusions:**

These results suggested that high level of HDAC6 expression in IgAN is correlated with renal dysfunction.

## Introduction

IgA nephropathy is one of the most common causes of chronic kidney disease (CKD) in China. A follow-up study found that 30–40% of patients with IgAN later developed into end-stage renal disease (ESRD) [1]. Though receiving kidney transplantation, about 15% of patients still suffer from transplant renal failure due to recurrent IgAN [2]. It is obvious that IgAN poses considerable threats and challenges to public health if not well controlled. Therefore, it is necessary to identify the renal function characteristics associated with IgAN in order to evaluate the illness severity and judge the prognosis in this disease.

Over the past 20 years, significant progresses have been made to help us understanding the pathogenesis of IgAN. A changed pattern of IgA1 glycosylation has been identified as a potential mechanism in IgAN for nearly 20 years [3], but the underlying mechanisms and prognosis factors of IgAN remains incompletely understood. Our previous studies found that CKD can be regulated by epigenetic modifications [4,5]. As far as we know, epigenetics mainly include three mechanisms: DNA methylation, histone modifications, and noncoding RNAs (ncRNAs) [6]. Recent studies have also demonstrated that histone acetylation and deacetylation act important roles in acute and chronic kidney diseases [7–11]. Acetylation and deacetylation are mainly catalyzed by histone acetyltransferases and histone deacetylase (HDACs) [12,13]. HDACs are a group of enzymes that mediate the removal of acetyl groups from target proteins including histones or non-histones, leading to condensation of chromatin structure and suppressing gene expression, eventually regulating their functions. There are four classes HDACs based on homology to their yeast analogs: class I HDACs (HDAC1, 2, 3, and 8), class II HDACs are divided into two subclasses, class IIa HDACs (HDAC4, 5, 7, and 9) and class IIb HDACs (HDAC6 and 10), respectively, class III (SIRT1-7), and class IV HDAC (HDAC11) [12–14].

HDAC6, existing in the cytoplasm, is unique among the 18 isoforms of the HDACs [15,16]. The most typical substrates of HDAC6 include α-Tubulin, heat shock protein 90, cortactin, stress granules and peroxiredoxin [16–19]. HDAC6 mediates many important biological processes, including cellular proliferation and apoptosis, transcription, immune response, and protein degradation [20–22]. There are an increasing number of evidences indicating that the expression and activity of HDAC6 are increased in many kidney diseases. The level of HDAC6 expression was increased in a mouse model of acute kidney injury (AKI) induced by cisplatin, treatment with TA reduced the serum creatinine and blood urea nitrogen (BUN) levels, increased the level of acetyl histone H3 expression, and decreased the level of HDAC6 expression in the injured kidney [23]. Moreover, HDAC6 plays an active role in lupus nephritis [24,25], treatment with a novel HDAC6-selective inhibitor CKD-506 can improve renal outcomes [26]. Furthermore, growing evidence has demonstrated that HDAC6 plays an important role in polycystic kidney disease [27]. These data indicated that HDAC6 is associated with the development and progression of various kidney diseases. However, it remains unclear whether HDAC6 also participated in the progression of IgA nephropathy.

To gain a better understanding of the correlation between HDAC6 and IgAN, we conducted two centers, retrospective study of 46 patients of biopsy-confirmed IgAN and 7 patients of normal control. We determined the level of renal HDAC6 expression in IgAN patients and compared it with patients' clinical characteristics.

## Materials and methods

### Clinical sample collection and ethics statement

The present study was performed at the Department of Nephrology of the Shanghai East Hospital Affiliated to Tongji University School of Medicine and Department of Nephrology of Shanghai General Hospital Affiliated to Shanghai Jiao Tong University School of Medicine. Patients who were diagnosed with IgAN by renal biopsy and patients who had renal carcinoma and underwent nephrectomy from January 2016 to September 2019 were enrolled. The demographic data, physical measurements, and laboratory measurements were collected from the electronic medical record system. This study was approved by the Human Research Ethics Committee of the Shanghai East Hospital Affiliated to Tongji University School of Medicine (Ethical Approval number: 2020-021) and the Human Research Ethics Committee of Shanghai General Hospital Affiliated to Shanghai Jiao Tong University School of Medicine (Ethical Approval number: 2021KY014). Written informed consent was obtained from all participants. And we have obtained the registration number from Chinese Clinical Trial Register (ChiCTR): ChiCTR 2000030422.

### Data collection

We collected general and clinical information, including patients sex, age, body mass index (BMI), blood pressure, glucose, glycosylated hemoglobin, hemoglobin, urea, creatinine, uric acid, cystatin C, triglyceride (TG), total cholesterol (TC), high density lipoprotein (HDL), low density lipoprotein (LDL), electrolyte, homocysteine, β2-microglobulin, complement C3, complement C4, 24 h urinary protein quantification, microalbuminuria, immunoglobulin A, and immunoglobulin kappa light chain from 46 patients with IgAN and 7 patients who had renal carcinoma and underwent nephrectomy. We also collected medical history of the patients, including the health behaviors (e.g., smoking and alcohol consumption), and therapies. The kidney biopsy specimens were collected before received these therapies.

BMI was calculated using the standard formula of weight (kg)/height (m^2^). Blood pressure was measured by a trained health care staff using an electronic sphygmomanometer. The blood pressure was the average of measurement three times with 5 min intervals among them. The eGFR was calculated using the formula CKD-Epidemiology Collaboration Equation (CKD-EPI) [28].

### Morphological studies of kidney

Formalin-fixed kidney tissues were imbedded in paraffin and prepared in 3-μm-thick sections. Masson trichrome staining was performed according to the protocol provided by manufacture (Sigma, St. Louis, MO). The Masson trichrome positive staining area (blue color) was quantitatively measured by using Image Pro-Plus software (Media-Cybernetics, Silver Spring, MD, USA) by drawing a line around the perimeter of positive staining area, and the average ratio to each microscopic field (200×) was calculated and graphed.

### Immunohistochemical staining

Immunohistochemical staining was performed according to the procedure described in our previous studies [5]. Tissue sections were immunostained with primary antibodies against anti-HDAC6 (#7612, Cell Signaling Technology, Danvers, MA) and anti-acetyl histone H3 (#9649, Cell Signaling Technology, Danvers, MA). For quantitative assessment, the HDAC6 positive area was measured by Image J software (National Institutes of Health, Bethesda, MD), and the average ratio to each microscope filed was calculated and graphed (200×). The acetyl histone H3 positive staining cells were counted in 10 high-power filed and calculated the average numbers of each microscope filed (200×).

### Statistical analysis

IBM SPSS V.20.0 was used for all statistical analyses. Distribution of variables was assessed by Kolmogorov-Smirnov test, and homogeneity of variance was evaluated by the Levene test. The clinical and demographic data were compared between IgAN patients and control group using the Student's *t* test, difference in proportion between the two groups were using χ^2^ test. Normally distributed data were expressed as the means ± SD, categorical variables are indicated in percentages. Univariate analysis of variance was used to analyze the difference among the groups in normal distribution data. Correlation test analysis was used to study the relationship between HDAC6 and clinical parameters, Pearson correlation analysis for bivariate normal distribution and Spearman correlation analysis for non-normal distribution data. Using factors identified by the univariate linear regression, multiple linear regression analysis was performed to determine the association of HDAC6 with various independent variables. Collinearity diagnostics were used to confirm whether the predictors are highly intercorrelated. The column and scatter charts were drawing using Excel 2010 (Microsoft Corp). For all analyses, *P* value < 0.05 were considered to be statistically significant.

## Results

### Clinical characteristics of patients with IgAN and normal controls

In total, 46 patients with IgAN qualified for analysis, there are 25 males and 21 females. 7 patients who had renal carcinoma and underwent nephrectomy were served as the normal control, including 3 males and 4 females. The clinical and demographic characteristics of patients are demonstrated in Table 1. Compared with the control group, the IgAN patients had higher level of creatinine than the normal control (*p* = 0.036). Moreover, the IgAN patients had lower level of magnesium ion than the control group (*p* < 0.001). BMI, hemoglobin, TG, TC, HDL, LDL, and uric acid were not significantly different between the IgAN patients and normal controls (*p* > 0.05).

**Table 1.** Comparison of general clinical data between IgA nephropathy and control group.

Variables	IgA nephropathy group (*n* = 46)	Control group (*n* = 7)	*p* Value
General data			
Male	25 (54.3%)	3 (42.9%)	0.634
Age	35.13 ± 11.44	69.86 ± 6.20	<0.001
BMI (kg/m^2^)	24.26 ± 3.90	23.28 ± 2.72	0.526
Systolic pressure (mmHg)	134.17 ± 20.33	138.14 ± 14.02	0.621
Diastolic pressure (mmHg)	89.80 ± 13.50	80.86 ± 6.99	0.093
Smoking	4 (8.7%)	2 (28.6%)	0.416
Drinking	3 (6.5%)	1 (14.3%)	0.748
Laboratory examination			
Glucose (mmol/L)	5.02 ± 0.82	5.96 ± 1.73	0.246
Glycosylated hemoglobin (%)	5.39 ± 0.45	6.00 ± 0.72	0.070
Hemoglobin (g/L)	133.37 ± 19.32	126.43 ± 16.39	0.372
Albumin (g/L)	38.68 ± 5.91	42.44 ± 4.65	0.114
Urea (mmol/L)	6.29 ± 2.49	5.42 ± 1.52	0.379
Creatinine (μmol/L)	109.80 ± 49.32	68.57 ± 24.25	0.036
Uric acid (μmol/L)	365.91 ± 94.17	300.86 ± 76.77	0.088
Cystatin C (mg/L)	1.27 ± 0.53	1.09 ± 0.34	0.387
Triglyceride (mmol/L)	1.74 ± 1.25	1.64 ± 0.31	0.896
Cholesterol (mmol/L)	4.73 ± 1.04	4.37 ± 1.00	0.571
HDL (mmol/L)	1.21 ± 0.39	1.04 ± 0.30	0.471
LDL (mmol/L)	3.16 ± 0.94	2.88 ± 0.97	0.619
K^+^ (mmol/L)	4.06 ± 0.56	4.38 ± 0.23	0.152
Na^+^ (mmol/L)	141.41 ± 2.22	141.43 ± 1.90	0.982
Cl^-^ (mmol/L)	103.23 ± 2.32	101.61 ± 1.89	0.087
Ca^2+^ (mmol/L)	2.23 ± 0.11	2.22 ± 0.19	0.928
P^-^ (mmol/L)	1.13 ± 0.19	1.20 ± 0.05	0.078
Fe^3+^ (μmol/L)	16.35 ± 6.56	12.93 ± 3.32	0.133
Mg^2+^ (mmol/L)	0.84 ± 0.06	0.94 ± 0.03	0.001
Therapy			
Beta blockers	3 (6.5%)	0 (0.0%)	0.787
ACEI/ARB	44 (95.7%)	1 (14.3%)	<0.001
Statins	12 (26.1%)	0 (0.0%)	0.282
Febuxostat	3 (6.5%)	0 (0.0%)	0.787
CCB	11 (23.9%)	1 (14.3%)	0.690
Glucocorticoid	24 (52.2%)	0 (0.0%)	0.026

Normally distributed data were expressed as the means ± SD, categorical variables are indicated in percentages, *p* value < 0.05 were considered to be statistically significant.

ACEI: angiotensin converting enzyme inhibitor; ARB: angiotensin receptor antagonist; BMI: body mass index; HDL: high density lipoprotein; LDL: low density lipoprotein; CCB: calcium channel blockers.

### The level of HDAC6 and acetyl histone H3 expression in IgAN patients

**Figure 1.**
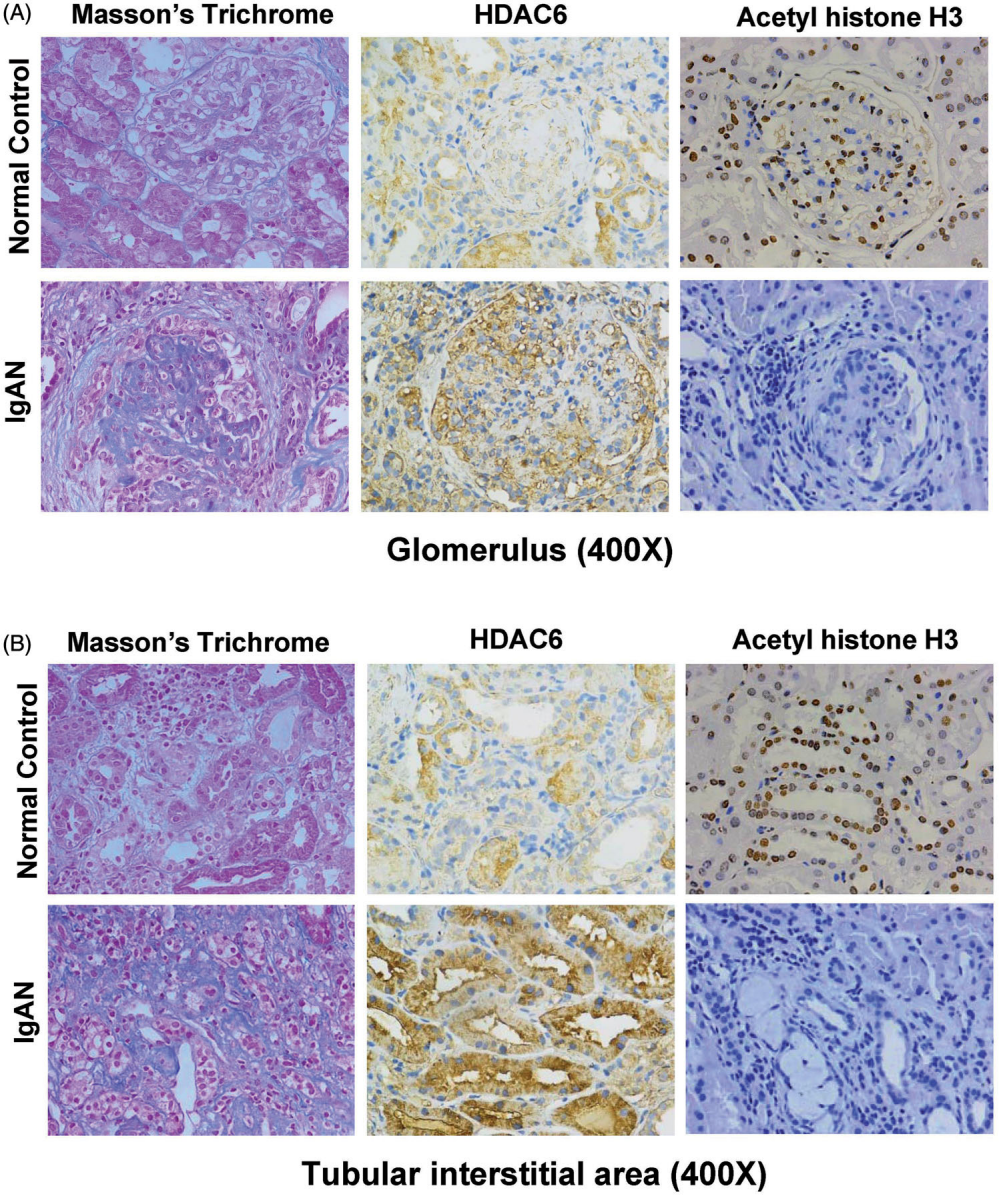
The level of HDAC6 and acetyl histone H3 expression in IgAN patients. (A) Representative glomerulus micrographs show the expression and localization of HDAC6 and acetyl histone H3 in human kidney biopsy specimens with IgAN and specimens of normal renal cortex tissue that was not affected by the tumor from patients with renal carcinoma (400×). (B) Representative tubular interstitial micrographs show the expression and localization of HDAC6 and acetyl histone H3 in human kidney biopsy specimens with IgAN and specimens of normal renal cortex tissue that was not affected by the tumor from patients with renal carcinoma (400×). IgAN: IgA nephropathy; normal control, specimens of normal renal cortex tissue that was not affected by the tumor from patients with renal carcinoma

To evaluate whether HDAC6 expressed in the kidney of IgAN, we collected renal biopsy specimens from IgAN patients and specimens of normal renal cortex tissue that was not affected by the tumor from patients with renal carcinoma. In all of the IgAN tissue samples, HDAC6 was found to localize specifically in tubular epithelial cells and glomeruli (Figure 1(A,B)). The level of HDAC6 expression in IgAN patients was significantly higher compared to that in control group. HDAC6 can induce deacetylation of histones, such as histone H3, so we further assessed the level of acetyl histone H3 expression in the kidneys. Our research indicated that acetyl histone H3 was predominantly localized in the glomeruli and renal tubule of the normal kidney tissues, while its expression level was remarkably decreased in IgAN patients (Figure 1(A,B)). Taken together, these results indicated that the level of HDAC6 expression was higher in the tissues of IgAN patients than in control samples, while the level of acetyl histone H3 expression was decreased in the tissues of IgAN patients.

### Relationship between oxford classification in renal biopsy of IgAN and clinical data

**Figure 2.**
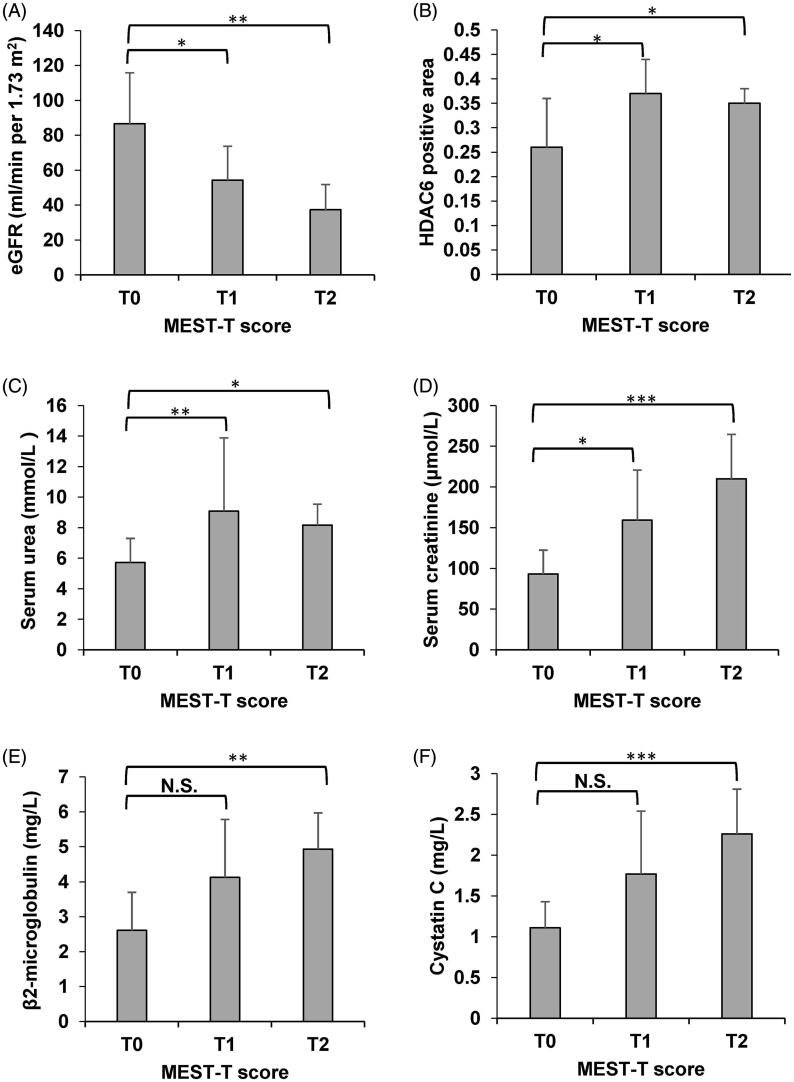
Relationship of the presence of the T lesion of the Oxford Classification with key clinical variables. Comparison of eGFR (A), HDAC6 positive area (B), serum urea (C), serum creatinine (D), β2-microglobulin (E), and cystatin C (F) among T0, T1 and T2 lesions. T0 group (*n* = 36), T1 group (*n* = 6), and T2 group (*n* = 3). Dta were presented as mean ± SD. *p* value < 0.05 were considered to be statistically significant. *<0.05, **<0.01, ***<0.001 vs T0.

The Oxford Classification of IgAN, proposed by the working group of the International IgA Nephropathy Network and the Renal Pathology Society, also known as MEST score, has been considered to be of significant value to predict the prognosis of IgAN, including mesangial hypercellularity (M), endocapillary hypercellularity (E), segmental glomerulosclerosis(S), tubular atrophy/interstitial fibrosis(T) [29]. Based on Oxford Classification, the relationship between Oxford Classification in renal biopsy of IgAN and clinical data is shown in Table 2. There was statistical difference in the index of HDL between M0 and M1 (*p* < 0.05), there were statistical difference in the indexes of albumin and Ca^2+^ between E0 and E1 (*p* < 0.05), there were statistical difference in the indexes of albumin, serum creatinine, cystatin C, and Ca^2+^ between S0 and S1 (*p* < 0.05), there were statistical difference in the indexes of eGFR, serum urea, serum creatinine, serum uric acid and HDAC6 positive area between T0 and T1 (*p* < 0.05), there were statistical difference in the indexes of eGFR, serum urea, serum creatinine, serum uric acid, cystatin C, cholesterol, high density lipoprotein, low density lipoprotein, Fe^3+^, β2-microglobulin, and HDAC6 positive area between T0 and T2 (*p* < 0.05). As for the tubular atrophy/interstitial fibrosis, this study revealed that the HDAC6 positive area of T1 score and T2 score patients were statistically higher than that of T0 score patients (*p* < 0.05), and associated with lower eGFR (Figure 2).

**Table 2.** Relationship between Oxford classification in renal biopsy of IgAN and clinical data.

Variables	Mesangial hypercellularity score		Endocapillary hypercellularity score		Segmental glomerular sclerosis score		Tubular atrophy and interstitial fibrosis score		
	M0 (*n* = 6)	M1 (*n* = 39)	E0 (*n* = 39)	E1 (*n* = 6)	S0 (*n* = 16)	S1 (*n* = 29)	T0 (*n* = 36)	T1 (*n* = 6)	T2 (*n* = 3)
BMI (kg/m^2^)	25.18 ± 2.09	24.19 ± 4.13	24.30 ± 4.15	24.45 ± 2.03	23.45 ± 3.11	24.80 ± 4.27	24.10 ± 3.69	25.59 ± 5.89	24.50 ± 2.46
Systolic pressure (mmHg)	132.67 ± 16.93	134.77 ± 21.11	133.39 ± 19.70	141.67 ± 25.63	135.13 ± 23.63	134.14 ± 18.90	133.11 ± 20.03	146.00 ± 21.95	128.00 ± 21.79
Diastolic pressure (mmHg)	88.83 ± 14.80	90.21 ± 13.57	89.49 ± 13.09	93.50 ± 17.32	88.44 ± 13.70	90.90 ± 13.65	88.61 ± 11.80	101.17 ± 17.59	84.67 ± 19.66
Glucose (mmol/L)	5.05 ± 0.60	5.02 ± 0.87	5.05 ± 0.88	4.89 ± 0.37	4.93 ± 0.35	5.08 ± 0.99	5.07 ± 0.92	4.92 ± 0.28	4.75 ± 0.27
Hemoglobin (g/L)	135.50 ± 18.87	133.15 ± 19.84	135.23 ± 19.57	122.00 ± 16.14	133.81 ± 18.49	133.28 ± 20.39	135.25 ± 18.26	131.17 ± 25.90	116.67 ± 19.40
Albumin (g/L)	39.78 ± 6.32	38.87 ± 5.51	39.83 ± 5.20	33.60 ± 5.10**	41.42 ± 4.38	37.66 ± 5.75*	39.41 ± 5.10	37.38 ± 8.97	37.20 ± 2.50
eGFR (ml/min per 1.73 m^2^)	83.08 ± 46.67	78.43 ± 49.07	80.62 ± 31.39	68.83 ± 31.47	85.77 ± 23.10	75.34 ± 34.84	86.64 ± 29.21	54.33 ± 19.34*	37.33 ± 14.47**
Urea (mmol/L)	7.41 ± 1.78	6.17 ± 2.57	6.34 ± 2.57	6.29 ± 2.25	5.80 ± 1.62	6.62 ± 2.86	5.72 ± 1.58	9.08 ± 4.81**	8.17 ± 1.37*
Creatinine (μmol/L)	118.77 ± 69.30	108.41 ± 47.26	107.45 ± 46.29	125.00 ± 72.72	92.06 ± 25.21	119.57 ± 57.36*	93.21 ± 29.22	159.17 ± 61.58*	210.00 ± 54.56***
Uric acid (μmol/L)	390.50 ± 88.85	363.97 ± 96.06	369.90 ± 93.76	352.00 ± 107.74	359.63 ± 67.78	371.86 ± 107.46	348.72 ± 82.83	439.33 ± 129.35*	449.33 ± 55.81*
Cystatin C (mg/L)	1.35 ± 0.72	1.26 ± 0.51	1.26 ± 0.54	1.34 ± 0.51	1.09 ± 0.27	1.37 ± 0.61*	1.11 ± 0.32	1.77 ± 0.77	2.26 ± 0.55***
Triglyceride (mmol/L)	1.44 ± 0.34	1.81 ± 0.34	1.80 ± 1.33	1.48 ± 0.66	1.87 ± 1.68	1.69 ± 0.96	1.81 ± 1.36	1.29 ± 0.47	2.02 ± 1.05
Cholesterol (mmol/L)	4.80 ± 0.78	4.70 ± 1.09	4.73 ± 1.06	4.63 ± 1.04	4.74 ± 1.04	4.70 ± 1.07	4.88 ± 1.05	4.33 ± 0.93	3.60 ± 0.10***
HDL (mmol/L)	1.01 ± 0.19	1.23 ± 0.40*	1.20 ± 0.41	1.21 ± 0.24	1.23 ± 0.42	1.18 ± 0.37	1.24 ± 0.39	1.15 ± 0.29	0.76 ± 0.08***
LDL (mmol/L)	3.63 ± 0.76	3.07 ± 0.95	3.15 ± 0.95	3.10 ± 0.94	3.12 ± 1.04	3.16 ± 0.90	3.25 ± 0.98	2.91 ± 0.77	2.37 ± 0.20***
K^+^ (mmol/L)	3.62 ± 1.34	4.13 ± 0.32	4.07 ± 0.60	4.03 ± 0.28	4.09 ± 0.36	4.05 ± 0.66	4.05 ± 0.62	4.18 ± 0.34	4.01 ± 0.17
Na^+^ (mmol/L)	142.47 ± 1.51	141.31 ± 2.28	141.38 ± 2.26	142.00 ± 2.00	142.30 ± 1.99	141.00 ± 2.24	141.55 ± 2.21	141.17 ± 1.17	141.00 ± 4.36
Cl^-^ (mmol/L)	103.68 ± 3.65	103.09 ± 2.09	103.08 ± 2.41	103.72 ± 1.50	102.45 ± 2.39	103.56 ± 2.21	102.91 ± 2.16	103.53 ± 2.60	105.47 ± 3.11
Ca^2+^ (mmol/L)	2.20 ± 0.09	2.24 ± 0.11	2.25 ± 0.10	2.13 ± 0.11*	2.30 ± 0.09	2.20 ± 0.10**	2.25 ± 0.09	2.15 ± 0.16	2.16 ± 0.07
P^-^ (mmol/L)	1.13 ± 0.29	1.12 ± 0.18	1.12 ± 0.20	1.14 ± 0.10	1.16 ± 0.21	1.10 ± 0.19	1.13 ± 0.19	1.03 ± 0.26	1.20 ± 0.08
Fe^3+^ (μmol/L)	14.53 ± 2.39	16.35 ± 6.84	16.18 ± 6.36	16.08 ± 8.17	15.19 ± 5.22	16.69 ± 7.19	17.32 ± 6.28	13.45 ± 6.99	9.63 ± 3.61*
Mg^2+^ (mmol/L)	0.84 ± 0.08	0.85 ± 0.06	0.84 ± 0.07	0.86 ± 0.05	0.84 ± 0.06	0.85 ± 0.07	0.84 ± 0.07	0.83 ± 0.04	0.88 ± 0.07
β2-microglobulin (mg/L)	2.54 ± 1.93	3.05 ± 1.26	2.97 ± 1.29	3.06 ± 1.96	2.57 ± 1.34	3.15 ± 1.36	2.61 ± 1.09	4.13 ± 1.65	4.93 ± 1.04**
beta blockers	0 (0%)	3 (7.69%)	2 (5.13%)	1 (16.67%)	0 (100%)	3 (10.34%)	1 (2.78%)	1 (16.67%)	1 (33.33%)
ACEI/ARB	6 (100%)	37 (94.87%)	37 (94.87%)	6 (100%)	15 (93.75%)	28 (96.55%)	34 (94.44%)	6 (100%)	3 (100%)
Statins	1 (16.67%)	11 (28.21%)	10 (25.64%)	2 (33.33%)	2 (12.5%)	10 (34.48%)	10 (27.78%)	1 (16.67%)	1 (33.33%)
Febuxostat	1 (16.67%)	2 (5.13%)	3 (7.69%)	0 (0%)	1 (6.25%)	2 (6.90%)	2 (5.56%)	1 (16.67%)	0 (0%)
CCB	2 (33.33%)	9 (23.08%)	10 (25.64%)	1 (16.67%)	3 (18.75%)	8 (27.59%)	6 (16.67%)	3 (50%)	2 (66.67%)
Masson trichrome positive area	0.36 ± 0.16	0.34 ± 0.13	0.35 ± 0.12	0.33 ± 0.24	0.32 ± 0.08	0.36 ± 0.16	0.32 ± 0.09	0.46 ± 0.16	0.43 ± 0.37
HDAC6 positive area	0.29 ± 0.09	0.28 ± 0.11	0.28 ± 0.11	0.31 ± 0.07	0.27 ± 0.10	0.29 ± 0.11	0.26 ± 0.10	0.37 ± 0.07*	0.35 ± 0.03*
Acetyl histone H3 positive cells	132 ± 84	125 ± 66	126 ± 70	130 ± 52	141 ± 73	118 ± 65	136 ± 68	88 ± 59	63 ± 21

Normally distributed data were expressed as the means ± SD, categorical variables are indicated in percentages, *p* value < 0.05 were considered to be statistically significant. M1 versus M0, E1 versus E0, S1 versus S0, T1 versus T0, T2 versus T0, *<0.05, **<0.01, ***<0.001.

ACEI: angiotensin converting enzyme inhibitor; ARB: angiotensin receptor antagonist; BMI: body mass index; CCB: calcium channel blockers; eGFR: estimated glomerular filtration rate; HDL: high density lipoprotein; LDL: low density lipoprotein.

### The expression of HDAC6 and acetyl histone H3 in various eGFR levels

**Figure 3.**
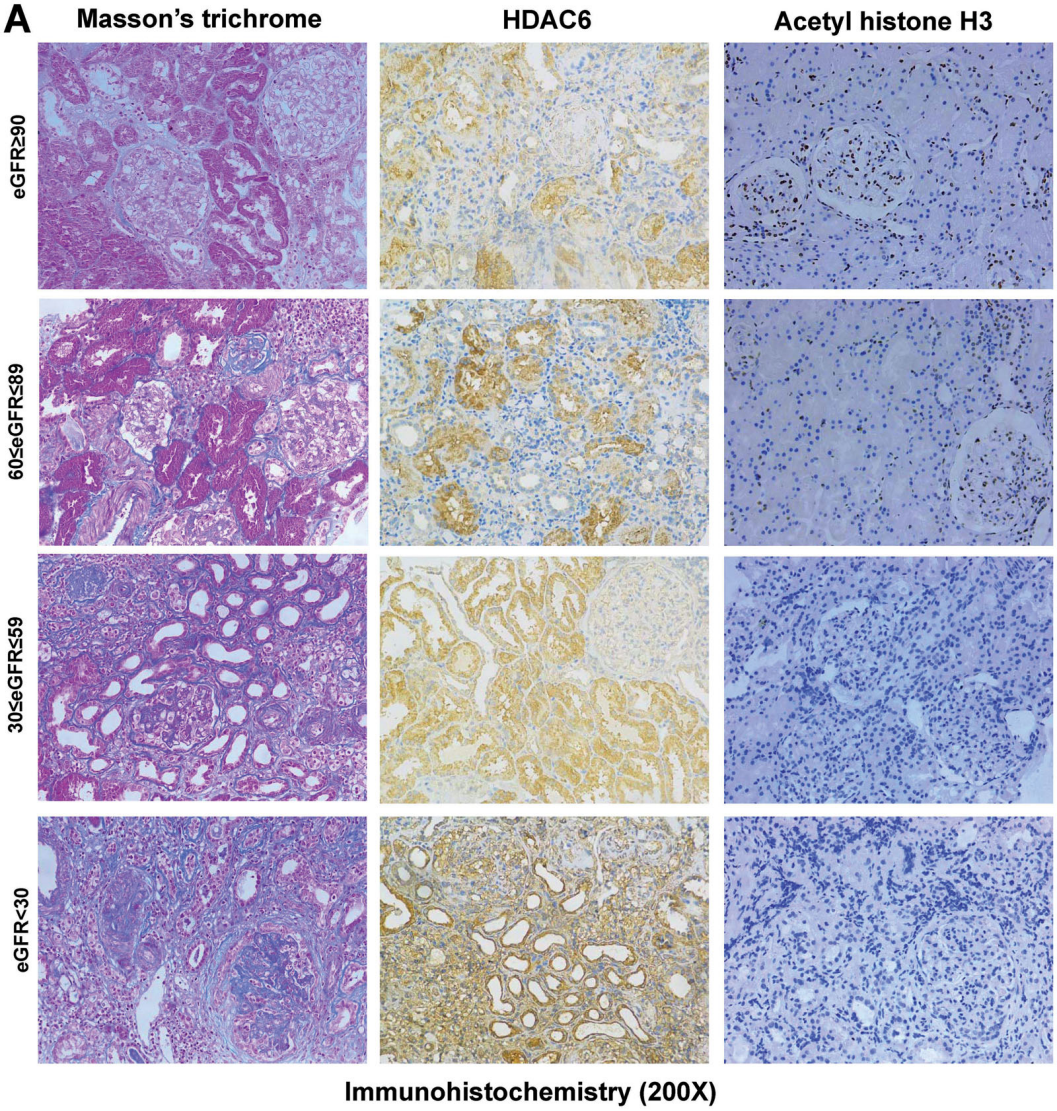
The expression of HDAC6 and acetyl histone H3 in various eGFR levels. We divided the subjects into four groups according to eGFR levels: group 1 (≥90 ml/min per 1.73 m^2^), group 2 (60–89 ml/min per 1.73 m^2^), group 3 (30–59 ml/min per 1.73 m^2^), group 4 (<30 ml/min per 1.73 m^2^). Representative micrographs show the expression and localization of HDAC6 and acetyl histone H3 in human kidney biopsy specimens with IgAN and specimens of normal renal cortex tissue that was not affected by the tumor from patients with renal carcinoma in various eGFR levels (200×).

**Figure 4.**
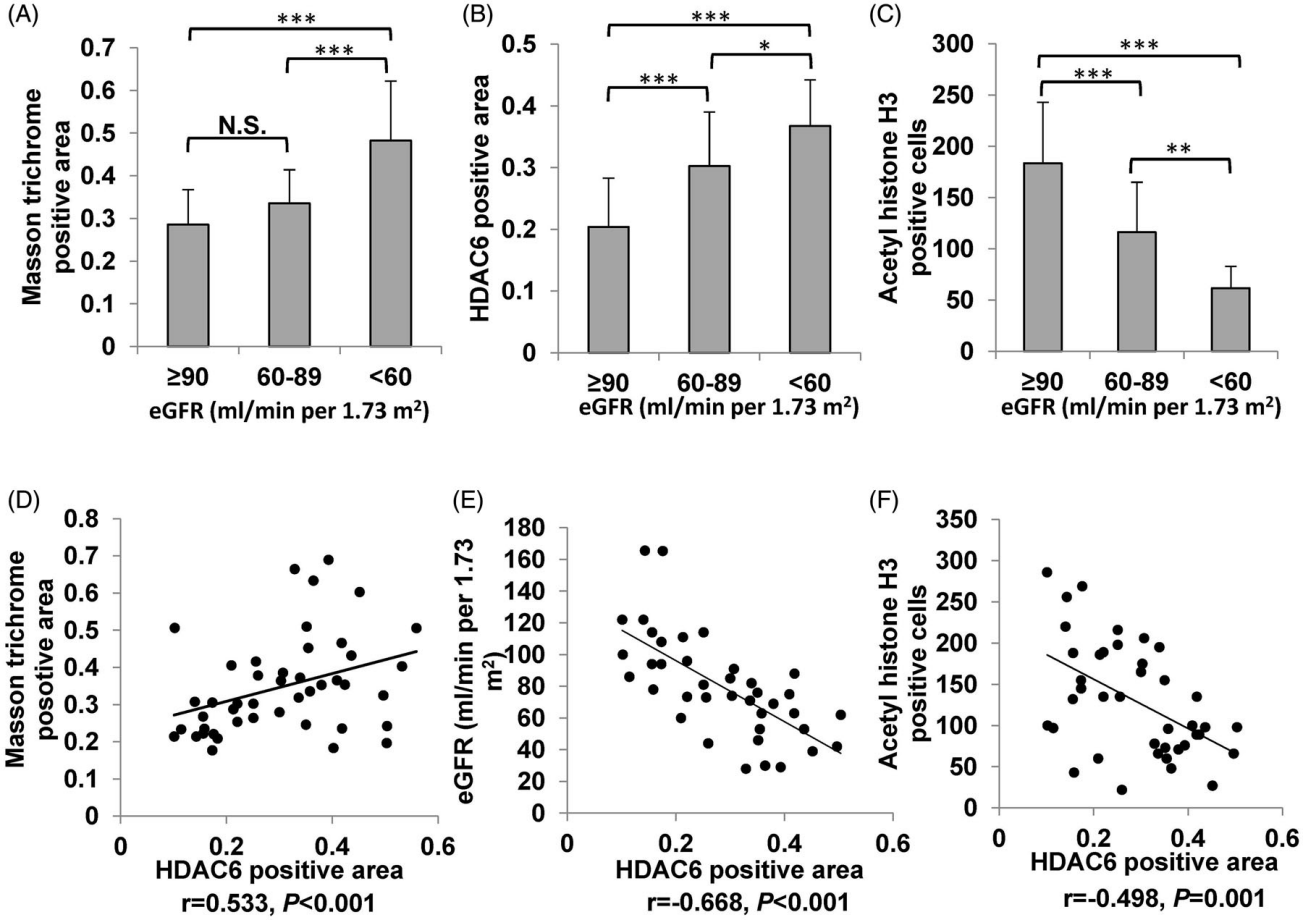
The relationships between HDAC6, acetyl histone H3 and eGFR. (A) The level of Masson trichrome staining positive area was quantitatively assessed by analysis of Masson trichrome staining in various eGFR levels. (B, C) The level of HDAC6 and acetyl histone H3 expression were quantitatively assessed by analysis of immunohistochemical staining in various eGFR levels. *p < 0.05, **p < 0.01, ***p < 0.001 versus each other. Scatter plots show significant correlation between the level of HDAC6 expression and Masson trichrome staining positive area (D), eGFR (E), and acetyl histone H3 positive cells (F). The Pearson correlation coefficient and *P* value are shown.

We divided the subjects into four groups according to eGFR levels: group 1 (≥90 ml/min per 1.73 m^2^), group 2 (60–89 ml/min per 1.73 m^2^), group 3 (30–59 ml/min per 1.73 m^2^), and group 4 (<30 ml/min per 1.73 m^2^). As shown in Figures 3 and 4(A–C), the level of HDAC6 expression were different in various eGFR levels, the expression of HDAC6 increased with the decreasing of eGFR level. The ratios of Masson trichrome staining positive area in IgAN patients with an eGFR <60 ml/min per 1.73 m^2^ (48.2%) was significantly higher than in patients with an eGFR of 60–89 ml/min per 1.73 m^2^ (33.8%, *p* < 0.001) and in patients with an eGFR ≥90 ml/min per 1.73 m^2^ (29.8%, *p* < 0.001). In addition, our research shown that the ratios of HDAC6 positive area in IgAN patients with an eGFR <60 ml/min per 1.73 m^2^ (36.7%) was higher than in patients with an eGFR of 60–89 ml/min per 1.73 m^2^ (30.4%, *p* = 0.04) and an eGFR ≥90 ml/min per 1.73 m^2^ (20.9%, *p* < 0.001). On the other hand, the level of acetyl histone H3 expression decreased with the decreasing of eGFR level. The numbers of acetyl histone H3 positive cells in IgAN patients with an eGFR <60 ml/min per 1.73 m^2^ (62, *p* < 0.001) and an eGFR of 60–89 ml/min per 1.73 m^2^ (116, *p* < 0.01) were lower than in patients with an eGFR ≥90 ml/min per 1.73 m^2^ (171). Collectively, this data suggested that with the deteriorates of the renal function in IgAN patients, the level of HDAC6 expression increases, but the level of acetyl histone H3 expression decreases.

### Correlation analysis of HDAC6, acetyl histone H3 expression levels and clinical parameters in IgAN patients

**Figure 5.**
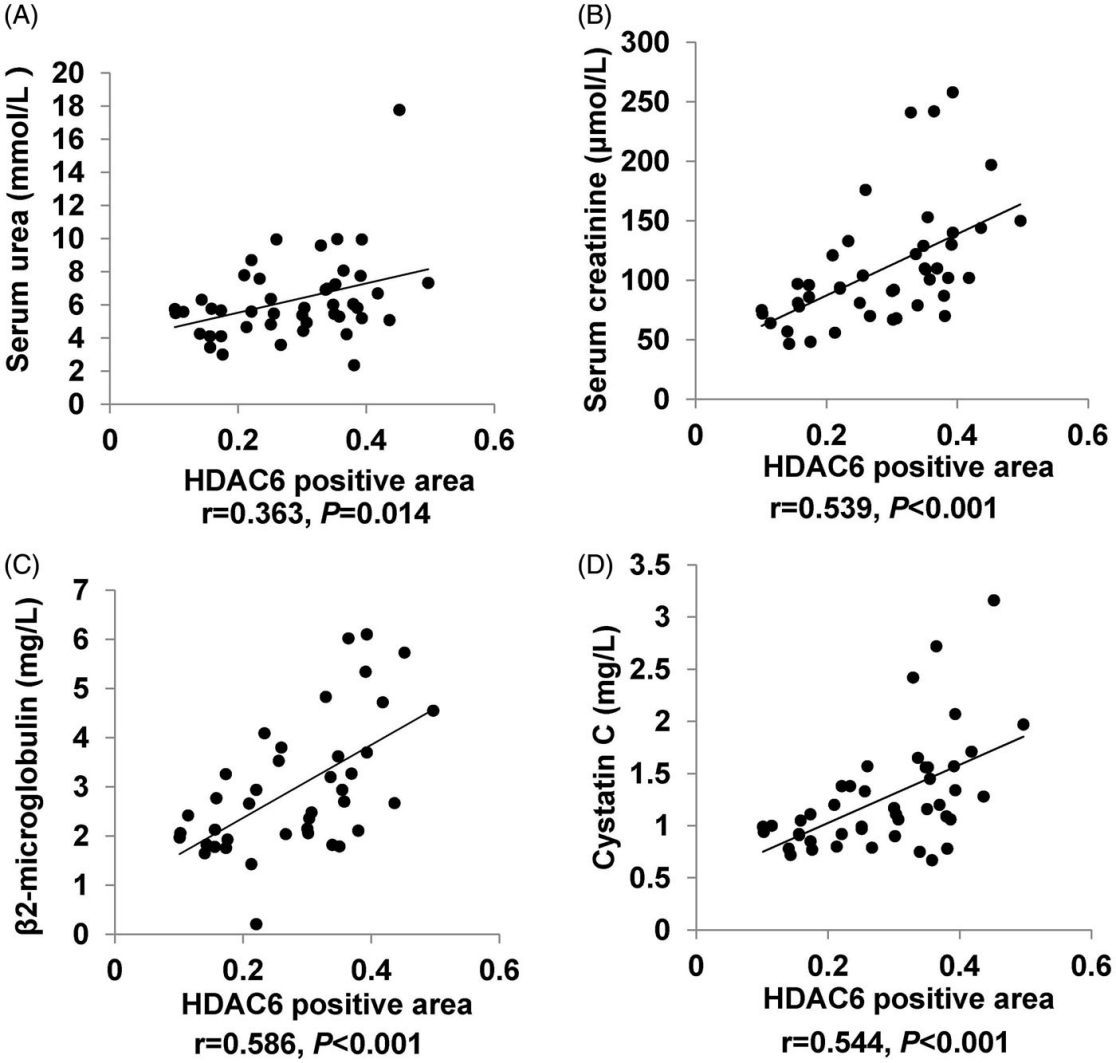
Correlation analysis of HDAC6 and clinical parameters in IgAN patients. Correlation analysis of HDAC6 and urea (A), creatinine (B), β2-microglobulin (C), and cystatin C (D) in IgAN patients. The Pearson correlation coefficient and *P* value are shown.

**Figure 6.**
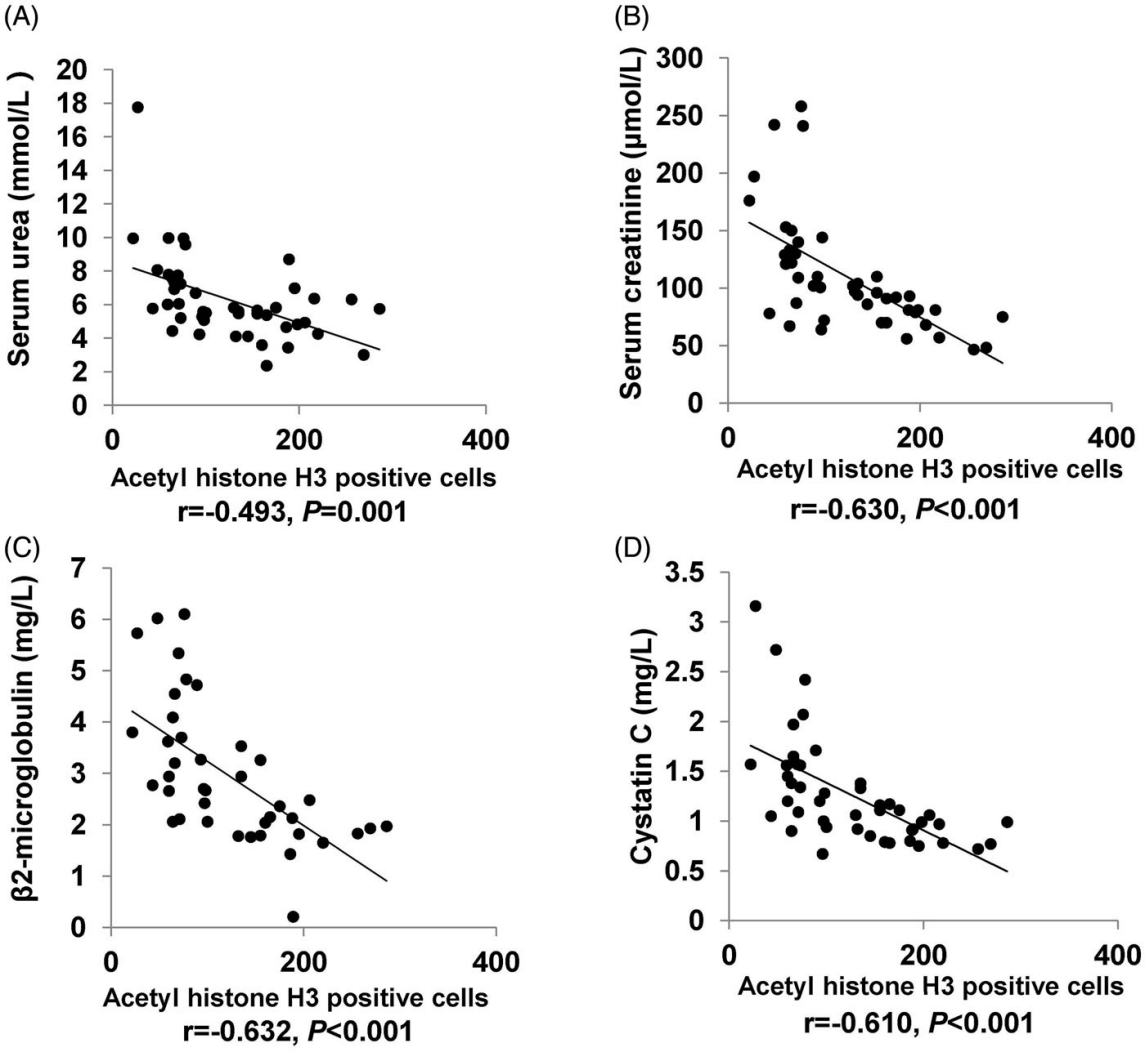
Correlation analysis of acetyl histone H3 and clinical parameters in IgAN patients. Correlation analysis of acetyl histone H3 and urea (A), creatinine (B), β2-microglobulin (C), and cystatin C (D) in IgAN patients. The Pearson correlation coefficient and *P* value are shown.

The correlative factors of HDAC6 in IgAN patients were determined using correlation test analysis. In present study, age, BMI, systolic pressure, diastolic pressure, glucose, urea, creatinine, uric acid, cystatin C, TG, TC, HDL, LDL, homocysteine, β2-microglobulin, C3, C4, 24 h urinary protein quantification, microalbuminuria, immunoglobulin A, and immunoglobulin kappa light chain were normally distributed data, so we used Pearson correlation test analysis to study the relationships among them, and the results were demonstrated in Table 3. The level of HDAC6 expression was negatively correlated with eGFR (*r* = −0.668, *p* < 0.001) and acetyl histone H3 (*r* = −0.498, *p* = 0.001) (Figure 4(E,F)). The level of HDAC6 expression was positively correlated with Masson trichrome staining positive area (*r* = 0.533, *p* < 0.001) (Figure 4(D)), urea (*r* = 0.363, *p* = 0.014), creatinine (*r* = 0.539, *p* < 0.001), β2-microglobulin (*r* = 0.586, *p* < 0.001) and cystatin C (*r* = 0.544, *p* < 0.001) (Figure 5). Moreover, acetyl histone H3 positive cells was negatively correlated with serum urea (*r* = −0.493, *p* = 0.001), serum creatinine (*r* = −0.630, *p* < 0.001), β2-microglobulin (*r* = −0.632, *p* < 0.001), and cystatin C (*r* = −0.610, *p* < 0.001) (Figure 6). Multiple linear regression analysis showed that HDAC6 was still positively correlated with cystatin C (*p* = 0.014), and negatively correlated with eGFR (*p* = 0.005). There was no obvious collinearity among these indicators (Table 4). Collectively, these data demonstrated that HDAC6 is tightly correlated with renal function in IgAN.

**Table 3.** Correlation analysis of HDAC6 levels and variables in IgAN patients.

Variables	*r*	*p*
Age	0.024	0.873
BMI (kg/m^2^)	0.056	0.713
Systolic pressure (mmHg)	−0.093	0.545
Diastolic pressure (mmHg)	0.066	0.668
Glucose (mmol/L)	0.139	0.370
eGFR (ml/min per 1.73 m^2^)	−0.668	<0.001***
Acetyl histone H3	−0.498	0.001***
Urea (mmol/L)	0.363	0.014*
Creatinine (μmol/L)	0.539	<0.001***
Uric acid (μmol/L)	0.095	0.534
Cystatin C (mg/L)	0.544	<0.001***
Triglyceride (mmol/L)	−0.146	0.344
Cholesterol (mmol/L)	−0.228	0.136
HDL (mmol/L)	−0.119	0.440
LDL (mmol/L)	−0.007	0.964
Homocysteine (μmol/L)	0.178	0.277
β2-microglobulin (mg/L)	0.586	<0.001***
Immunoglobulin A (g/L)	0.055	0.737
Immunoglobulin kappa light chain (g/L)	−0.194	0.295
C3 (g/L)	0.075	0.630
C4 (g/L)	0.271	0.075

BMI: body mass index; HDL: high density lipoprotein; LDL: low density lipoprotein; eGFR: estimated glomerular filtration rate.

*p* value <0.05 were considered to be statistically significant. *<0.05, ***<0.001 vs each other.

**Table 4.** The multiple linear regression for HDAC6 in IgAN patients.

Variable	Unstandardized coefficients		Standardized coefficients	*t* value	*p* Value	95.0% CI for B	Collinearity statistics	
	*B*	Std. error	β				Tolerance	VIF
eGFR (ml/min per 1.73 m^2^)	−0.003	0.001	−0.713	−3.378	0.005	−0.005 to −0.001	0.259	3.857
Cystatin C (mg/L)	0.168	0.060	0.850	2.814	0.014	0.04 to 0.296	0.127	7.901
β2-microglobulin (mg/L)	−0.033	0.022	−0.410	−1.495	0.157	−0.08 to 0.014	0.154	6.503
Diastolic pressure (mmHg)	−0.003	0.001	−0.418	−3.188	0.007	−0.005 to −0.001	0.674	1.485
glycosylated hemoglobin (%)	0.032	0.022	0.163	1.423	0.177	−0.016 to 0.079	0.878	1.139
Triglyceride (mmol/L)	−0.04	0.009	−0.617	−4.642	<0.001	−0.059 to −0.022	0.654	1.529
Immunoglobulin A (g/L)	−0.035	0.014	−0.447	−2.510	0.025	−0.064 to −0.005	0.364	2.750
Immunoglobulin kappa light chain (g/L)	0.0530	0.019	0.446	2.736	0.016	0.011 to 0.094	0.434	2.305

eGFR: estimated glomerular filtration rate; VIF: variance inflation factor.

*p* Value < 0.05 were considered to be statistically significant.

## Discussion

In this study, we found that HDAC6 was up-regulated in injured tubular epithelial cells and glomeruli in IgAN patients, and the level of acetyl histone H3 expression was decreased. There was statistical difference in the HDAC6 positive area among the different Oxford Classification. As for the tubular atrophy/interstitial fibrosis, this study revealed that the HDAC6 positive area of T1 score and T2 score patients were statistically higher than that of T0 score patients. Intriguingly, HDAC6 and acetyl histone H3 expression closely correlated with the disease progression in these patients. The expression of HDAC6 increased with the decreasing of eGFR levels, on the contrary, the expression of acetyl histone H3 decreased with the decreasing of eGFR levels. In addition, HDAC6 was positively correlated with urea, creatinine, cystatin C and β2-microglobulin, and negatively correlated with eGFR and acetyl histone H3 in IgAN patients. Taken together, this study demonstrated that the HDAC6 highly expressed in patients with IgAN is tightly correlated with renal dysfunction.

HDAC6, the class IIb deacetylase, has recently emerged as a critical cytokine in kidney diseases. A serial analysis of gene expression (SAGE) data for HDAC6 suggests that HDAC6 is minimally express in normal kidney tissues [30], however, the level of HDAC6 expression is up-regulated in various kidney diseases, such as autosomal dominant polycystic kidney disease (ADPKD) [31], lupus nephritis [32], and AKI [23]. In this study, we found that the level of HDAC6 expression was also increased in the kidney of IgAN patients. Moreover, our data indicated that the expression of HDAC6 in IgAN was negatively correlated with eGFR, and positively correlated with serum urea, serum creatinine, β2-microglobulin and cystatin C, five clinical parameters evaluate the renal function. Multiple linear regression analysis showed that HDAC6 was still positively correlated with cystatin C, and negatively correlated with eGFR. As far as we know, this is firstly demonstrated that the HDAC6 highly expressed in patients with IgA nephropathy is tightly correlated with renal dysfunction. How HDAC6 lead to the renal dysfunction in IgAN is currently unknown. To our knowledge, renal fibrosis constitutes a major health concern in IgAN, the major features of renal fibrosis include deposition of extracellular matrix components (ECM) and differentiation of different types of cells to myofibroblasts [33], eventually results in the decreased of glomerular filtration rate. Previous studies demonstrated that HDAC6 involved in renal fibrosis in animal models. A study showed that the expression of HDAC6 was increased in a mouse model of renal fibrosis, treatment with HDAC6 selective inhibitor or small interfering RNA against HDAC6 attenuated the renal fibrosis [34]. Shan et al. revealed that TGF-β1-induced EMT is accompanied by HDAC6-dependent deacetylation of α-Tubulin [35], inhibition of HDAC6 decreased TGF-β1-induced EMT markers and the formation of stress fibers. Choi et al. demonstrated that the fibrotic mechanism of HDAC6 was that HDAC6 involve in both regulation of epigenetic histone modification and promotion of phospho-Smad2/3 to Smad3 binding elements in fibrotic genes. However, our present study is a cross-sectional project, whether the HDAC6 could induce renal fibrosis in IgAN then lead to the renal dysfunction is still unknown. In the near future, we will perform the follow-up study to address this issue.

In the Oxford Classification, the pathological findings of M, E, S, and T were selected by univariate and multiple regression analyses, and the Cox proportional hazard regression model was additionally selected in 2016 [29,36,37]. At the same time, the updated Oxford Classification of IgAN recommends cellular/fibrocellular crescent formation (C) as a pathological predictor of renal outcomes of IgA nephropathy, which known as MEST-C score system, can provide a more comprehensive pathological prediction for the prognosis of IgAN [37]. In the current study, we did not use the C score because of the two centers used the 2009 Oxford Classification, also known as MEST score system, to classify the pathological of IgA nephropathy until 2018, so we don't have the data of C score in the year of 2016 and 2017. In this classification, each pathological finding can be evaluated individually as a split system, its usefulness has been widely accepted after the analyses conducted by a large number of validation studies [38–40], so we studied the relationship of the presence of the M, E, S, and T lesions of the Oxford Classification with key clinical variables respectively. Our study showed that the patients of M1 has more than that M0, the patients of E0 has more than that E1, and the patients of S0 has slightly less than S1, this is consistent with previous study, which is reported that the most common characteristic of IgAN on light microscopy is mesangial cell proliferation [41]. However, our study showed that the patients of T0 has more than that T1 and T2, this may be ascribed to that the current research is a cross-sectional study, the patient's prognostic outcome still needs further follow-up.

In addition, multiple studies revealed that lower serum albumin at the time of renal biopsy is indicator associated with poor prognosis of IgAN [42,43]. Similarly, our study also indicated that the level of serum albumin was lower in E1 and S1 compared with E0 and S0, respectively. One of the interesting findings of our investigation was that there was statistical difference in the indexes of eGFR, serum urea, serum creatinine, serum uric acid, cystatin C, and β2-microglobulin among T1, T2 and T0, but there were not significant association between M0 and M1, E0 and E1, S0 and S1. Among the MEST-C score system, it has been reported that the T score is the most valuable histological parameter, Oxford-T lesion was an independent risk factor for IgAN [36]. T lesion is not only a histomorphological characteristic of IgAN but is quite a final common pathway for multiple progressive kidney diseases [44,45]. A study showed that tubular atrophy and interstitial fibrosis were independent indicators of ESRD but not mesangial cell proliferation [46]. Therefore, we speculated that the mesangial cell proliferation is probably an initiating factor while injury of tubular cell and podocyte are the key elements during the progression of IgAN.

Considering that T score is a well-recognized independent predictive indicator of IgAN, it is worth to explore the possible risk factors associated with T. It is reported that tubulointerstitial injury of IgAN was related to p38 mitogen-activated protein kinase activity [47], serum matrix metalloproteinase-7 (MMP-7) level [48], trefoil factor 3 mRNA [49], and H-related proteins 5 (FHR-5) deposition [50]. In this study, our results showed that the HDAC6 positive area of T1 score and T2 score patients were statistically higher than that of T0 score patients, and associated with lower eGFR. In other words, patients with IgAN with high level of HDAC6 presented with more serious T lesions, which may imply that the expression of HDAC6 in patients of IgAN might influence the prognosis. Our recent study demonstrated that HDAC6 contribute to fibrosis by direct activation of renal interstitial fibroblast, HDAC6 involve in renal fibrosis by the activation of TGF-β1/Smad3 and EGFR signaling pathways in UUO model [51]. However, the current research is an observation study, no evidences came from IgAN animal models had been available. Therefore, the *in vivo* studies are needed to reveal the mechanisms that result in HDAC6 expression and its relationship with T lesions in IgAN in our future studies.

It is reported that high HDAC6 expression was an independent, poor prognostic factor in renal cell carcinoma (RCC) patients, and HDAC6 are considered to be a biomarker of RCCs prognostic and an indicator for RCC progression [52]. However, whether the expression of HDAC6 could be a biomarker in IgAN still unclearly. In the past decades, efforts have been taken toward the development of biomarkers for early prediction of disease progression in IgAN. For instance, several biomarkers in serum (such as galactose-deficient IgA1 [Gd-IgA1], fibroblast growth factor 23 [FGF-23], MMP-7, and FHR5) [48,53–55], and urine (such as angiotensinogen [AGT], kidney injury molecule 1 [KIM-1], and miRNA-200a) [56–58] were found in previous studies. In our study, we found that HDAC6 highly expressed in patients with IgA nephropathy is tightly correlated with renal dysfunction, these might suggest that the expression of HDAC6 could be a potential prognosis factor for IgAN.

Some limitations of the study should be acknowledged. First, because of our data were all from patients who underwent renal biopsy, some of patients with IgA nephropathy did not accepted biopsy for various reasons, thus, selection bias was present in the study. Second, since our study only have 46 human kidney biopsy specimens with IgAN and 7 specimens of normal renal cortex tissue that was not affected by the tumor from patients with renal carcinoma, the identified of the conclusions reached should be conducted by an investigation involving with a large number of cases. Third, we should perform the follow-up study to obtain more convincing data to address the correlation between HDAC6 expression and clinical outcomes in IgA nephropathy patients in the near future. If possible, we need to explore the effect of HDAC6 on the development of IgA nephropathy in animal models in our future studies. Nonetheless, our study exerted with strict exclusion criteria based on medical histories and laboratory results.

In conclusion, the results of this study identified that the expression of HDAC6 was increased in the IgA nephropathy. With the deterioration of renal function, the expression of HDAC6 persistently increased. These findings provide a novel insight into our understanding the relationship of HDAC6 in IgA nephropathy.

## Ethics statement

This study was approved by the Human Research Ethics Committee of the Shanghai East Hospital Affiliated to Tongji University School of Medicine and the Human Research Ethics Committee of Shanghai General Hospital Affiliated to Shanghai Jiao Tong University School of Medicine. Written informed consent was obtained from all participants. This research study was conducted in accordance with the guidelines of the Declaration of Helsinki.
